# Visualizing the trans-synaptic arrangement of synaptic proteins by expansion microscopy

**DOI:** 10.3389/fncel.2024.1328726

**Published:** 2024-02-29

**Authors:** Stefan Sachs, Sebastian Reinhard, Janna Eilts, Markus Sauer, Christian Werner

**Affiliations:** Department of Biotechnology and Biophysics, Biocenter, University of Würzburg, Würzburg, Germany

**Keywords:** synapse, expansion microscopy, trans-synaptic, nanocolumns, Airyscan, super-resolution microscopy

## Abstract

High fidelity synaptic neurotransmission in the millisecond range is provided by a defined structural arrangement of synaptic proteins. At the presynapse multi-epitope scaffolding proteins are organized spatially at release sites to guarantee optimal binding of neurotransmitters at receptor clusters. The organization of pre- and postsynaptic proteins in trans-synaptic nanocolumns would thus intuitively support efficient information transfer at the synapse. Visualization of these protein-dense regions as well as the minute size of protein-packed synaptic clefts remains, however, challenging. To enable efficient labeling of these protein complexes, we developed post-gelation immunolabeling expansion microscopy combined with Airyscan super-resolution microscopy. Using ~8-fold expanded samples, Airyscan enables multicolor fluorescence imaging with 20–40 nm spatial resolution. Post-immunolabeling of decrowded (expanded) samples provides increased labeling efficiency and allows the visualization of trans-synaptic nanocolumns. Our approach is ideally suited to investigate the pathological impact on nanocolumn arrangement e.g., in limbic encephalitis with autoantibodies targeting trans-synaptic leucine-rich glioma inactivated 1 protein (LGI1).

## 1 Introduction

Synaptic transmission at chemical synapses demands precise organization of proteins in subsynaptic domains and close coordination of synaptic proteins in the millisecond range facilitating the release and transfer of neurotransmitters to postsynaptic receptors (Holderith et al., 2012; Nakamura et al., 2015). Despite their relevance for efficient transmission, the ultrastructural layout of synaptic proteins and their interplay remains to be elucidated. The main challenge for multicolor visualization of synaptic proteins is the minute size of clusters and compaction of proteins which is inaccessible by conventional labeling approaches and standard fluorescence microscopy. In the last decade, super-resolution (SR) microscopy approaches were developed that could reveal protein organization at the nanometer scale forming the basis for millisecond processes indispensable for synaptic function and supporting the comprehension of pathophysiological mechanisms (Compans et al., 2016; Haselmann et al., 2018; Nosov et al., 2020; Werner et al., 2021; Lycas et al., 2022). Nanoscale clusters of AMPA receptors (α-amino-3-hydroxy-5-methyl-4-isoxazolepropionic acid receptor) in the postsynaptic compartment form the receiving modules whereas platforms for synaptic vesicle depletion are decorated by subsynaptic domains of active zone proteins Munc13-1 (mammalian uncoordinated homology 13, domain 1) and Rab3-interacting molecule (RIM; Tang et al., 2016; Sakamoto et al., 2018). To render synaptic transmission more efficient, postsynaptic receptors are aligned with presynaptic RIM clusters by trans-synaptic nanocolumns that are getting more pronounced following synaptic long-term potentiation (Tang et al., 2016). Ultrastructural rearrangement of nanocolumns supporting homeostatic plasticity is also present at inhibitory synapses and deregulation is implicated in pathology-related mechanisms (Crosby et al., 2019; Fukata et al., 2021b; Yang et al., 2021; Muttathukunnel et al., 2022). SR imaging by single-molecule localization microscopy provides 10–20 nm spatial resolution but demands careful sample preparation, the addition of photoswitching buffers, long-term drift stabilization and careful data handling. Three-dimensional and multicolor localization microscopy, although feasible in principle, remains challenging (Schucker et al., 2015; Lelek et al., 2021). In contrast, expansion microscopy provides easy access to SR imaging in three dimensions allowing the multi-target visualization of synaptic protein distribution in synaptic compartments (Gao et al., 2019; Gallagher and Zhao, 2021; Sarkar et al., 2022). As an additional advantage, post-immunolabeling of expanded gels reduces the linkage error between epitope and fluorophore, and increases epitope accessibility, while bulk (pan) labeling of the proteome creates detailed maps of proteins in the ultrastructural synaptic context (Zwettler et al., 2020; Sarkar et al., 2022).

**Figure 1:**
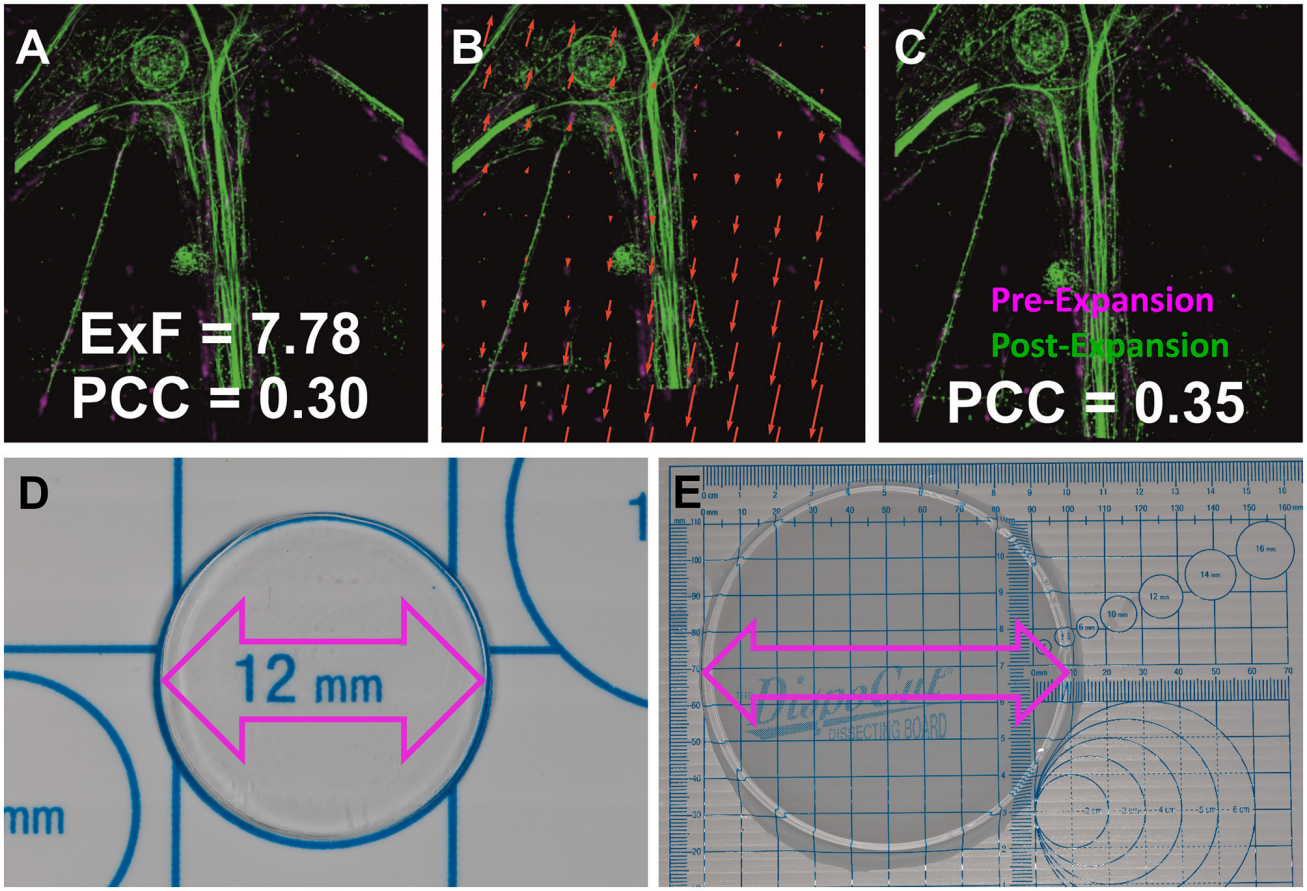
Determination of the structural ExF. **(A)** Pre- and post-expansion images of neurofilament L regions, depicting isotropic expansion in primary neurons. The similarity transformation involves four degrees of freedom: rotation, scaling, and translation in x, y. **(B)** Distortion map revealing vectorial shifts (red arrows) for optimal alignment from **(A–C)**. **(C)** Images aligned through an affine transformation, resulting in a slightly improved Pearson correlation coefficient (PCC). **(D)** Pre-expansion vs. **(E)** post-expansion gel photographs, indicating a notable increase from 12 mm to ~10 cm, corresponding to an expansion factor of ~8.3x.

Here we show that the combination of ~8-fold expansion microscopy (ExM; Figure 1) using the TREx-protocol and Airyscan SR imaging (ExM-AS) enables three-dimensional multicolor visualization of nanoscale trans-synaptic nanocolumns and analysis of their arrangement (Damstra et al., 2022). For nanocolumn analysis we developed a custom cross-correlation script that is available for the neuroscientific community under: https://github.com/super-resolution/Sachs-et-al-2023-supplement. Our analysis supports the nanocolumn arrangement of RIM 1/2 and PSD95 and shows that Munc13-1 is aligned with postsynaptic AMPA receptor subunits (GluA1). In addition, we co-visualize calcium channels and LGI1, a trans-synaptically secreted protein that links presynaptic ADAM23 and ADAM22 (metallopeptidase domain 23 and 22) across the synaptic cleft and is targeted by autoantibodies in limbic encephalitis (Sirerol-Piquer et al., 2006; Petit-Pedrol et al., 2018).

## 2 Results

**Figure 2:**
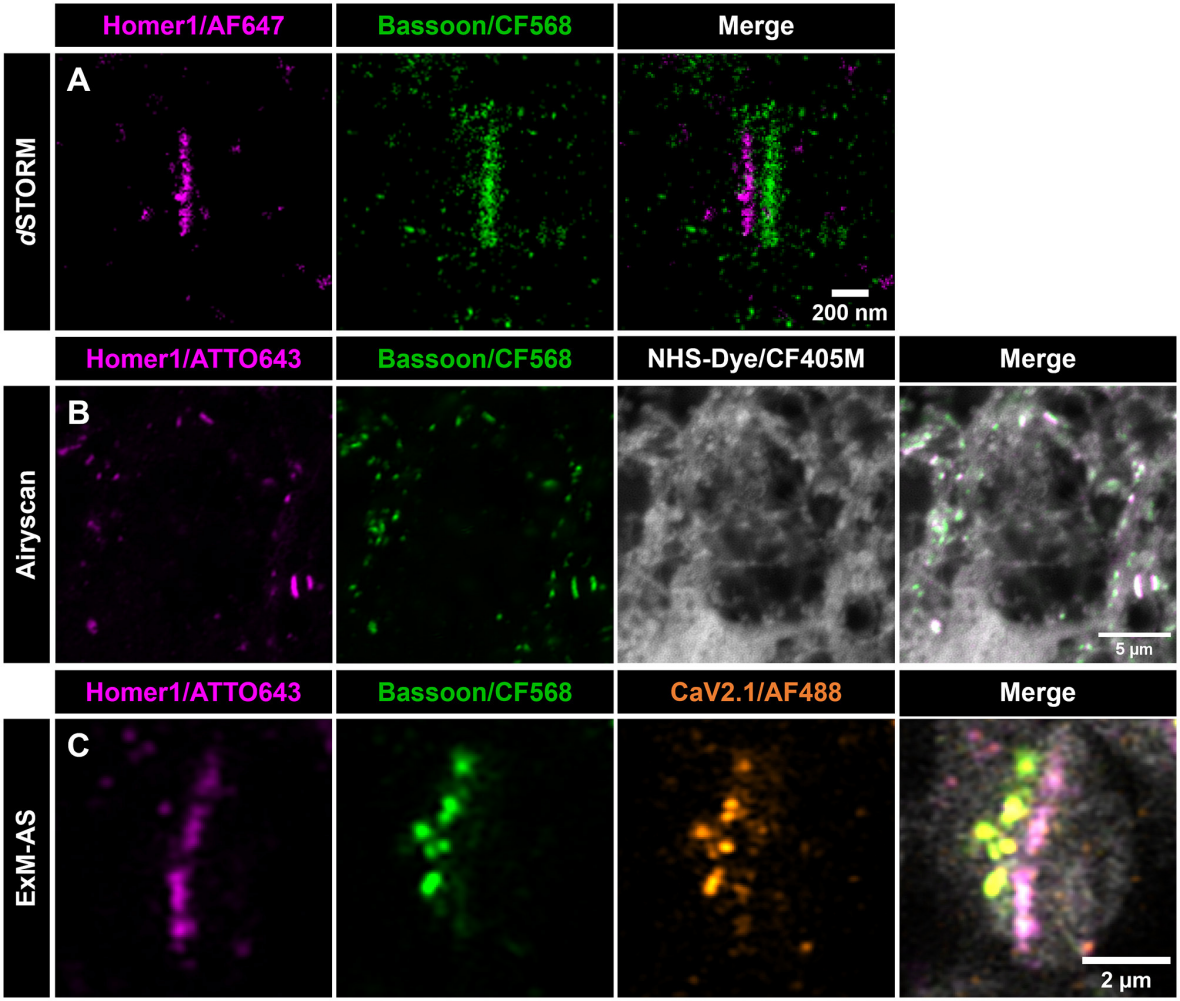
ExM-AS facilitates SR imaging of synapse organization. **(A)** *d*STORM images of a planar synapse, showing postsynaptic Homer1 (magenta) and presynaptic BSN (green). Scale bar: 200 nm. **(B)** Airyscan image (single z-stack slice) of non-expanded primary neurons labeled with Homer1 (magenta) and BSN (CF568, green) using immunolabeling. Neuronal proteome is pan-labeled with NHS-CF405M (gray). Scale bar: 5 μm. **(C)** Primary neurons immunolabeled with the same primary and secondary antibodies as in **(B)** for active zone (green) and post-synaptic side (magenta). P/Q voltage-dependent calcium channels CaV2.1 are labeled with AF488 (orange), and the neuronal proteome with NHS-CF405M (gray). Scale bar: 2 μm in ~8x expanded dimensions.

To demonstrate the benefits of the ExM-AS approach we first established conventional labeling of synaptic targets along with pan-NHS (N-Hydroxysuccinimid) staining of all proteins to visualize all accessible primary amines on synaptic proteins in hippocampal mouse neurons. Applying Airyscan microscopy, we detected presynaptic Bassoon (BSN) and postsynaptic Homer1 to uncover synaptic contact sites whereas pan-neuronal labeling provided additional information on the position of nuclei and layout of microtubules and actin filaments as well as the general orientation of neuronal axons and dendrites (Figure 2B and Supplementary Figure 1). To obtain information about the ultrastructural layout of the synaptic contact site, BSN opposing Homer1 separated by synaptic clefts can be visualized by dual-color direct stochastic optical reconstruction microscopy (*d*STORM; Figure 2A; Pauli et al., 2021). Applying ExM followed by post-expansion labeling and Airyscan imaging of BSN and Homer1 results in improved clarity of BSN signals due to the decrowding effect and higher resolution (Figure 2C). More specifically, Airyscan SR imaging reliably attains a 120 nm optical resolution (SR Mode), our findings regarding Expansion factor (ExF) determination (Figure 1) demonstrate ~8-fold sample expansion achieving optical resolution within the range of 20–40 nm.

Compared to conventional labeling for SR microscopy the post-labeling of decrowded synapses yields enhanced labeling efficiency by targeting so far inaccessible epitopes and visualization of individual clusters of BSN instead of bar-like shapes that are usually observed applying conventional immunolabeling and SR imaging without expansion of the sample (Siddig et al., 2020; Eilts et al., 2023). The visualization of distinct BSN clusters was also reported previously, but with a more complex protocol applying an iterative ExM process (Sarkar et al., 2022). In expanded specimens, pan-NHS labeling provides an ultrastructural context for the following immunolabeling approach by labeling the entire proteome e.g., intensifying protein dense regions at synaptic contact sites. The synaptic scaffolding proteins taken as reference here, align with the ultrastructural densities and provide clear orientation of the synapse (Figures 2B, C; M'Saad and Bewersdorf, 2020; Ons et al., 2022). However, a clear alignment of BSN and Homer1 clusters can be identified (mean PCC = 0.62 ± 0.11, 102 synapses from five independent neuronal cultures). Beyond visualizing the synaptic contact site, we co-immunolabeled P/Q type calcium channels (CaV2.1) exemplifying the multicolor feasibility of our established SR imaging approach (Figure 2C).

**Figure 3:**
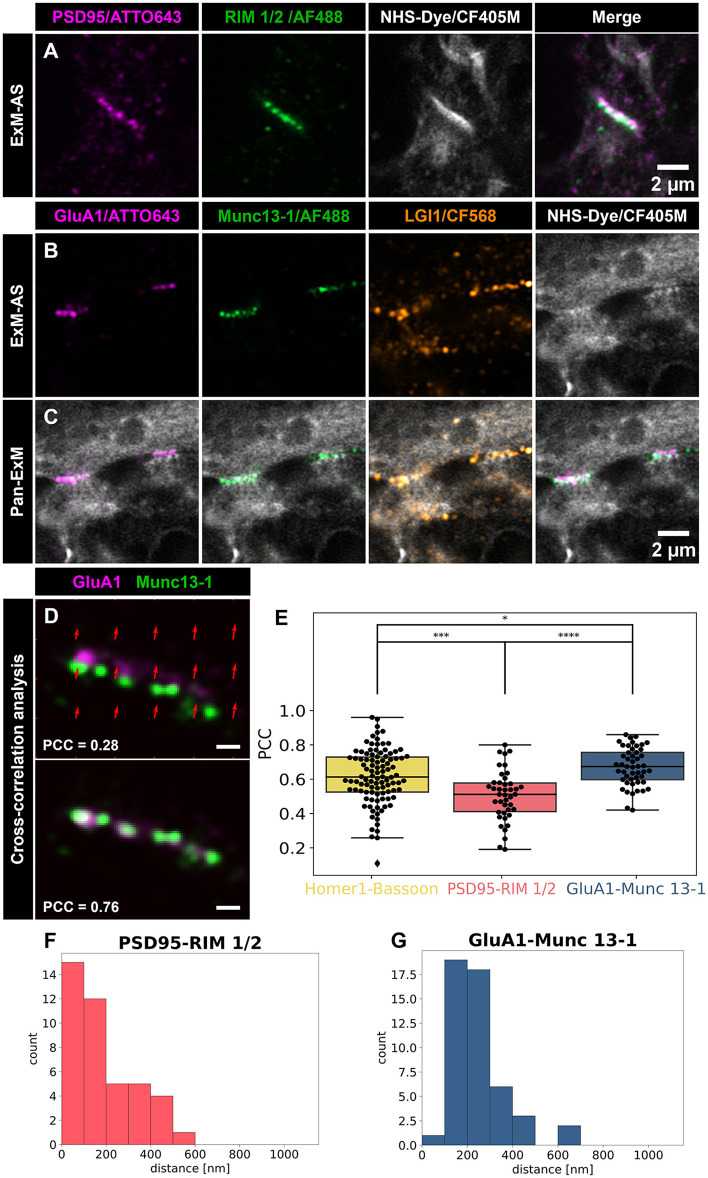
Quantification of nanocolumn arrangement at synapses resolved by ExM-AS. **(A)** Airyscan images of expanded hippocampal neurons with postsynaptic PSD95 (magenta) and presynaptic marker RIM 1/2 (green). Overview images include NHS-ester labeling (gray) for ultrastructural context. Scale bar: 2 μm. **(B)** Airyscan images of postsynaptic AMPAR subunit GluA1 (magenta), active zone marker Munc13-1 (green, AF488), and trans-synaptic LGI1 (orange, CF568). Synaptic proteome is labeled with NHS-CF405M (gray). **(C)** Overview Airyscan images showing frontal view of NHS-labeling and synaptic marker positions. Scale bar: 2 μm. **(D)** Example of GluA1 and Munc13-1 nanocolumn alignment, with distortion map and overlap. Scale bar: 0.5 μm. **(A–D)** Scale bars are in ~8x expanded dimensions and all samples were post-gelation immunolabeled. **(E)** Cross-correlation analysis for alignment: BSN and Homer1, RIM 1/2 and PSD95, Munc13-1 and GluA1. Box plots show the median, 25 and 75% quartile range. The whiskers indicate the 0 and 100% quartile range of the datapoints (black dots display measurements from individual synapses). Outliers, exceeding 1.5 × standard deviation are not taken into account. **(F)** Frequency distribution histogram of postsynaptic PSD95 and presynaptic RIM 1/2 signal distances. **(G)** Distances between postsynaptic GluA1 and presynaptic Munc13-1 signals **(F, G)**. Distance values are in ~8 fold expanded dimensions. *p < 0.05, ***p < 0.001, ****p < 0.00001.

We now reasoned that the application of our post-immunolabeling scheme and its combination with further optical super-resolving techniques might be beneficial for the visualization and analysis of recently discovered trans-synaptic nanocolumns (Tang et al., 2016; Fukata et al., 2021b). Next, we used ExM-AS with antibody-based post-expansion labeling of RIM 1/2 and PSD95 along with pan-neuronal labeling of all proteins applying NHS-ester CF405M to visualize nanocolumns (Figure 3A). Cross-correlation analysis indeed revealed a good trans-synaptic alignment of RIM 1/2 and PSD95 with mean Pearson values of 0.51 ± 0.15 (42 synapses from five independent neuronal cultures; Figure 3A, Supplementary Figures 2, 5).

Release sites of synaptic vesicles can be mapped by labeling of Munc13-1 and postsynaptic receptor fields were shown to be aligned with synaptic vesicle fusion sites (Tang et al., 2016; Sakamoto et al., 2018). To visualize the nanoscale alignment of sending and receiving ports of chemical synapses, we applied post-immunolabeling of Munc13-1 and GluA1 subunits of AMPA receptors, in combination with pan-NHS labeling (Figures 3B, C). The cross-correlation analysis of Munc13-1 and AMPA receptors revealed a strong alignment (mean PCC = 0.67 ± 0.11, 49 synapses from *n* = 6 independent neuronal cultures; Figure 3E). After testing for normal distribution of our data, we compared PCC values between the different groups by applying the parametric *t*-test showing that they differ significantly from each other, although they all show relatively strong PCC values. Cross-correlation of GluA1-Munc13-1 shows the highest PCC compared to PSD95/RIM 1/2 (*p* < 0.00001). Interestingly, the AMPA receptor signal overlapped with pan-NHS labeled postsynaptic protein densities and Munc13-1 signal mapped well to presynaptic protein dense clusters (Figures 3B–D). Using the NHS labeling signals, with reduced linkage error, we determined the distance between the pre- and post-synapse to ~318 nm corresponding to a size of the synaptic cleft of ~40 nm in the non-expanded state (Supplementary Figures 6C, D). From selected immunostainings of synaptic proteins we calculated distances between synaptic partners to evaluate binding performance of antibodies on decrowded synapses and provide information on relative localization of the nanocolumn pairs at synaptic regions (Figures 3F, G). For visualizing a trans-synaptic protein we co-immunolabeled LGI1, a secreted protein that binds presynaptic ADAM23 and links AMPA-receptors via interaction with postsynaptic ADAM22 (Fukata et al., 2006, 2010; Sirerol-Piquer et al., 2006).

**Figure 4:**
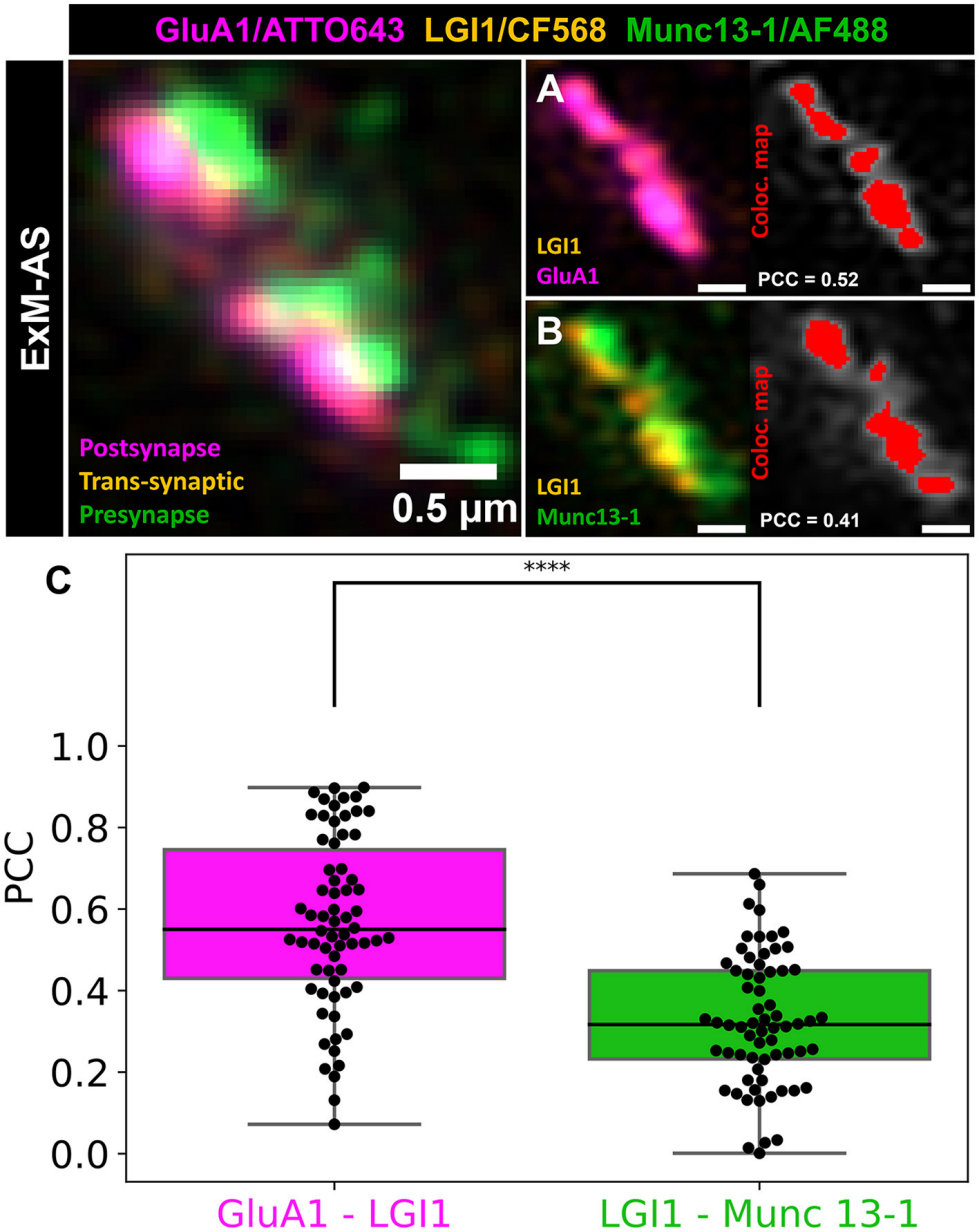
Trans-synaptic LGI1 colocalization with presynaptic Munc13-1 and postsynaptic GluA1. **(A)** Colocalization example of trans-synaptic LGI1 (orange) and postsynaptic GluA1 subunit (magenta). **(B)** Colocalization example of trans-synaptic LGI1 (orange) and presynaptic Munc13-1 (green). **(A, B)** Colocalization map (Coloc. map) is shown in red. Scale bars: 0.5 μm in ~8x expanded dimensions. **(C)** Colocalization analysis of trans-synaptic LGI1 with postsynaptic GluA1 clusters shown in the magenta box plot. The green box plot illustrates the colocalization analysis of LGI1 with presynaptic Munc13-1. Box plots illustrates the median, 25 and 75% quartile range. The whiskers indicate the 0 and 100% quartile range of the datapoints (black dots represent measurements from individual synapses). Outliers, exceeding 1.5 × standard deviation are not taken into account. ****p < 0.00001.

Colocalization analysis of LGI1 with Munc13-1 and GluA1 revealed good overlap with these pre- and postsynaptic structures indicating localization inside synaptic clefts. Interestingly, we observed higher Pearson values (mean PCC = 0.57 ± 0.21) for colocalization with postsynaptic AMPA receptor subunits (GluA1), than with presynaptic active zone Munc13-1 (mean PCC = 0.33 ± 0.16; *p* < 0.00001, *t*-test for normal distributed data validated by Shapiro-Wilk test; Figure 4). The majority of LGI1 signal mapped to the synaptic contact site between pre- and postsynaptic compartments and was closely associated with nanocolumns formed between Munc13-1 and GluA1 (Figures 3D, E).

## 3 Discussion

In the last decade SR microscopy approaches facilitated the investigation of the ultrastructural layout of neuronal synapses with so far unmatched detail (Schermelleh et al., 2019). Providing 10–20 nm resolution, localization microscopy was one of the first approaches that mapped synaptic receptors into subsynaptic domains and described the layout of several synaptic proteins respective to the synaptic cleft as well as the discovery of so called trans-synaptic nanocolumns (Dani et al., 2010; Compans et al., 2016; Tang et al., 2016). However, localization microscopy requires skilled experts building and maintaining complex optical systems, drift-stable setups, fluorophores with special biophysical characteristics and careful data handling procedures (Lelek et al., 2021). With the advent of ExM SR imaging of synapses was achieved on conventional confocal microscopy setups by physically expanding the sample in a hydrogel (Chen et al., 2015). Furthermore, the resolution can be enhanced by combing ExM with other optical based SR techniques, tweaking the monomer composition, as well as using iterative expansion steps (Schucker et al., 2015; Chang et al., 2017; Zwettler et al., 2020; Sarkar et al., 2022).

Our approach combines single-step ~8x sample expansion [Figure 1, Ten-fold Robust Expansion Microscopy; TREx protocol (Damstra et al., 2022)] with Airyscan SR imaging to reveal layout of synaptic proteins in the ultrastructural context. Combination of ExM with Airyscan imaging has been demonstrated in earlier studies, however both reports used pre-expansion labeling with higher linkage errors and without the positive effect of synaptic proteins decrowding. These protocols applied Proteinase K treatment that can potentially reduce the signal in concert with interaction of the fluorophores with radical starters (Wang et al., 2020; Gaudreau-Lapierre et al., 2021). In our approach presented here, post-labeling of expanded gels reduces the linkage error and minimizes signal loss during gelation and expansion steps along with improved antigen accessibility and increase labeling density. By reducing the linkage error, fluorophores are closer to the target protein and can reveal single nanodomains where higher linkage error approaches such as pre-gelation immunolabeling extend the expanded signal resulting in the appearance of bar-shaped structures. In comparison to conventional immunolabeled synapses, nanodomains of presynaptic active zone proteins or postsynaptic receptors and scaffolding proteins can be identified by applying ExM-AS (Figure 3). Increasing the expansion factor by iterative expansion procedures in combination with SR techniques might reveal additional subsynaptic organization principles pinpointing how vesicles, calcium channels and vesicular (v-)—and transmembrane (t-) soluble N-ethylmaleimide-sensitive-factor attachment receptor (SNARE) proteins are oriented with respect to nanocolumns (Chang et al., 2017; Sarkar et al., 2022). Indeed, some synapses show protein dense presynaptic regions with nanodomains connected by gaps suggesting space available for synaptic vesicle fusion sites (Figures 3B, C and Supplementary Figure 6). In agreement, recent EM-tomography revealed that 20% of trans-synaptic nanocolumns are linked to synaptic vesicles and future experiments may elucidate how synaptic vesicles and associated SNARE proteins are linked to trans-synaptic nanocolumns. Here, electron microscopy (EM) tomography was performed on high pressure frozen samples following freeze substitution providing excellent contrast, structural preservation and resolution for ultrastructure of protein densities but without additional immunostaining of specific proteins (Reese and Cole, 2023). In comparison, our ExM-AS combination creates ultrastructural context by NHS-labeling of the synaptic proteome inside PFA fixed expanded specimen combined with efficient post-labeling of decrowded synaptic regions with minor linkage-errors. Our approach allows identification of the organization of specific synaptic proteins by multicolor, three- dimensional fluorescence microscopy inside this ultrastructural context and protocols can be performed within 3–4 days compared to more complex and time-consuming EM approaches. Still, immunolabeling in EM can be performed by gold-conjugated secondary antibodies or by choosing embedding reagents like LR White compatible with fluorescence microscopy. However, combination of EM with immunolabeling can come at the cost of ultrastructural contrast and protocols as well as data processing require expert knowledge.

EM was the first method to reveal a clear ultrastructural context of synapses visualizing synaptic vesicles and protein dense regions like postsynaptic densities (Whittaker and Gray, 1962). In expanded specimen, pan-staining with NHS-dye conjugates achieves an ultrastructural context by targeting all accessible primary amines on available proteins along with multicolor visualization synaptic ultrastructure in three dimensions (M'Saad and Bewersdorf, 2020). In our experiments, pan-NHS labeling revealed protein dense structures representing synaptic contact sites and revealed that trans-synaptic proteins are indeed localized to synaptic clefts. Synaptic clefts measured ~318 nm in expanded dimension yielding ~40 nm distance across the cleft assuming an expansion factor of ~8x (Supplementary Figures 6D, E). A combination of pan-NHS labeling and immunolabeling of LGI1, a trans-synaptic protein linking presynaptic ADAM23 and postsynaptic ADAM22 showed that this trans-synaptic protein is closely associated with nanocolumns of Munc13-1 (median PCC = 0.32) and GluA1 (median PCC = 0.55) and is concentrated at the synaptic cleft (Figures 3B, C). It would be interesting to see where autoantibodies targeting LGI1 in limbic encephalitis will bind and if they can manipulate the integrity of nanocolumn arrangement.

In conclusion, our combination of SR approaches will facilitate the understanding of trans-synaptic nanocolumn ultrastructure by identifying the alignment of classical nanocolumn partners along with possible new nanocolumn proteins e.g., isoforms of neurexin and neuroligin at excitatory and inhibitory synapses (Dean and Dresbach, 2006). Increased resolution and reduced linkage error will allow investigation of their connection to other essential molecular players supporting synaptic efficacy like synaptic vesicles, SNARE complexes and synaptic receptors. Finally, ExM-AS can be advantageously used to improve our understanding of pathological alterations in limbic encephalitis or epilepsy where trans-synaptic LGI1 proteins are targeted by autoantibodies or are mutated, respectively (Zhou et al., 2009; Petit-Pedrol et al., 2018; Fukata et al., 2021a).

## 4 Materials and methods

### 4.1 ExM-experiments chemicals

Reagents for ExM experiments were purchased from Sigma-Aldrich, including acrylamide (AAm; A4058), N, N'methylenebis (acrylamide; BIS; M1533), sodium acrylate (SA; 408220), sodium chloride (NaCl; S5886), Tris- (hydroxymethyl)-aminomethane (Tris; 1.08382), ammonium persulfate (APS; A3678), and N, N, N', N'-tetramethylethylenediamine (TEMED; T7024). APS and TEMED were prepared as 10% (w/v) stock solutions in ddH_2_O. Additionally, sodium dodecyl sulfate (SDS; AM9820) was obtained from Invitrogen.

### 4.2 Hippocampal neuron preparation

Isolation of primary neurons: E18 C57BL/6 mice were used for primary hippocampal neuron isolation under approval from Bavarian state authorities. Hippocampal tissue underwent digestion in 0.25% trypsin-EDTA (ThermoFisher, 25300054) at 37°C for 15 min, followed by two washes in a solution of 1x Hanks' balanced salt solution (HBSS; ThermoFisher, 88284), 250 μl Gentamycin (Sigma, G1397). Trituration of hippocampi with varied pipette pore sizes preceded seeding onto poly-D-lysine (PDL; Sigma, P6407) coated coverslips. Specifically, 40,000 neurons were plated on each 12 mm PDL-coated coverslip (1 mg/ml, 1 h at RT, washed twice with ddH_2_O). Neurons were cultured in Neurobasal™ (ThermoFisher, A3582901) medium including 1:50 B27 Plus supplement (ThermoFisher, A3582801) and 1:100 Glutamax (ThermoFisher, 35050061) for 14 days at 37°C, 5% CO_2_, with 50% medium replacement weekly. Fixation utilized 4% methanol-free paraformaldehyde (PFA; ThermoFisher, 289036) for 15 min at RT, followed by two washes with 1x phosphate-buffered saline (PBS; Sigma; D1408).

### 4.3 Neuron fixation, permeabilization, and protein crosslinking

Neuronal cells were fixed with 4% PFA for 15 min, followed by three rinses in 1x PBS. Permeabilization was achieved with 0.2% Triton™ X-100 (ThermoFisher, 28314) in 1x PBS, followed by three additional washes. Subsequently, cells were crosslinked overnight at 37°C using a solution of 4% PFA and 30% acrylamide (AAm) in 1x PBS.

### 4.4 Gelation, denaturation, and post-gelation immunostaining

Monomer solutions comprising 1.1 M sodium acrylate (SA), 2 M acrylamide (AAm), 0.009% N, N'methylenebis (acrylamide; BIS), and 1x PBS were introduced to neurons under nitrogen. Polymerization was initiated with 10% TEMED and 10% APS, and gels were incubated for 1.5 h at RT in a nitrogen-filled, humidified chamber. Gels were detached, heat-denatured in denaturation buffer (200 mM SDS, 200 mM NaCl, 50 mM Tris) at 95°C for 1 h, and then cooled to RT, following four washes, each 15 min with 1x PBS to remove denaturation solution. Gel size was subsequently increased to a 2-3x expansion. Primary antibodies underwent overnight incubation in a blocking solution with 5% bovine serum albumin (BSA) dissolved in 1x PBS. Gels were washed four times with 0.1% TWEEN 20 (PBST) for 20 min. Secondary antibodies were added, and incubated at 37°C for 3 h (Supplementary Table 1). After immunolabeling, samples were washed four times with 0.1% PBST for 20 min using a spinning wheel at RT.

### 4.5 Pan-ExM NHS staining

After immunolabeling, the entire proteome was labeled with 40 μg/ml NHS-CF405M (Biotium, 92111) (Supplementary Table 1) in a solution of 100 mM NaHCO_3_ dissolved in 1x PBS for 1.5 h. Subsequently, gels were transferred to a Petri dish and immersed in ddH_2_O. 6–7 exchanges with ddH_2_O and the gels achieved full expansion to their maximum sizes.

### 4.6 ExM sample mounting

Once the expansion process was completed, the gels were mounted on one-well chambers coated with PDL (1 mg/ml), to minimize drift. These imaging chambers contain 0.17 mm thick glasses (Cellvis, C1-1.5H-N).

### 4.7 Airyscan imaging

The LSM 900 laser scanning microscope features multiple used objectives, including Plan-Apochromat 63x/1.40 oil immersion and C-Apochromat 40x/1.2 water-immersion. Diode lasers include 30 mW 405 nm, 30 mW 488 nm, 25 mW 639 nm, and 25 mW 561 nm diode pumped solid state (DPSS) lasers. Detection capabilities include two GaAsP PMTs for regular confocal imaging and a 32 GaAsP PMT array for Airyscan detection. Utilizing the LSM 900 with Airyscan 2 technology in SR imaging mode achieved a spatial resolution of 120 nm in non-expanded samples.

For dye selection, the integrated dye presets of ZEN 2 blue software (version 3.5) from Zeiss were employed. Standard strength mode of 3D Airyscan processing was applied to all captured images. During Airyscan image processing, channel alignment corrected for chromatic aberration. As part of this process, 100 nm Tetraspecks^TM^ (Invitrogen, T7279) were diluted 1:1000 in ddH_2_O and incubated for 30 min with an unstained, fully expanded hydrogel. The processed non-expanded Airyscan data had a pixel size of 99 nm, while all processed expanded ExM-AS data for illustrations and quantifications had a pixel size of 41.2 nm.

### 4.8 Immunostaining of non-expanded neurons*, d*STORM imaging, and *d*STORM data processing

For illustrating non-expanded samples (Supplementary Figure 1) and two-color *d*STORM experiments (Figure 2A), primary neurons were fixed with 4% PFA for 10 min at RT. After fixation, cells were washed with 1x PBS and permeabilized with 0.2% Triton^TM^ X-100 (ThermoFisher, 28314) for 10 min at RT. Subsequently, they were blocked with 5% BSA in 1x PBS for 1 h at RT. Primary antibodies (Supplementary Table 1) were added and incubated for 1 h at RT, followed by three washing steps with 0.1% PBST and a 1 h incubation with secondary antibodies. An additional post-fixation step with 4% PFA for 15 min at RT was performed. Samples were stored in 1x PBS at 4°C until imaging.

During *d*STORM imaging, a reducing agent was present in the imaging buffer to facilitate reversible photoswitching of fluorophores, composed of 100 mM β-mercaptoethylamine (Sigma-Aldrich, M6500) in 1x PBS (pH 7.7).

Two-color *d*STORM measurements were conducted using an inverted fluorescence wide-field microscope (Olympus, IX-71) equipped with an oil-immersion objective (APON 60/1.49; Olympus) and a nose-piece stage (Olympus, IX2-NPS) to minimize vibrations and drift. A 639 nm laser (Coherent, OPSL, Genesis MX STM Series) and a dichroic mirror (Chroma, ZT405/514/635rpc) were used for Alexa Fluor 647 excitation. In the detection path, another dichroic mirror (Chroma, 630 DCXR customized) and a bandpass filter (Semrock, 679/41 BrightLine series) were placed in front of two separate electron-multiplying charge-coupled device (EMCCD) cameras (Andor, iXon Ultra 897) to capture emitted light. For CF568, a 558 nm laser, a dichroic mirror (Chroma, FF410/504/582/669), and a 607/70 bandpass filter (Semrock, Brightline) were used. The two channels were imaged sequentially with an exposure time of 20 ms, capturing 15,000 frames using Total Internal Reflection Fluorescence (TIRF) illumination and an irradiation intensity of 3–5 kW/cm^2^. Single molecule localization data were reconstructed with a pixel size of 10 nm using the open-source software rapidSTORM 3.3. Chromatic aberration was corrected by imaging 0.1 μm fluorescent microspheres (ThermoFisher, T7279) after each measurement and creating an alignment matrix with the ImageJ plugin bUnwarpJ (Schucker et al., 2015). The matrix was then applied to the reconstructed two-color images.

### 4.9 Antibodies and NHS-dye conjugation

To conjugate the goat anti-rabbit secondary antibody (Invitrogen, 31212) with NHS-ATTO643 (ATTO-TEC, AD 643-31), Zeba^TM^ Spin Desalting Columns with a 40 K MWCO (ThermoFisher, 87766) were employed. The coupling process followed the instructions provided by the manufacturer, using a reaction medium of 100 mM NaHCO_3_ (ThermoFisher, 144558) and a 7-fold molar excess of the NHS-dye. The incubation took place in a dark room at RT for 3 h. The labeled antibodies were subsequently purified using Zeba^TM^ Spin Desalting Columns with 0.02% NaN_3_ (Sigma, S-8032) in 1x PBS to eliminate any non-bound dye. To determine the antibody concentration (0.6 mg/ml) and degree of labeling (DOL, 1.6) of ATTO643, absorption measurements were taken at 280 and 643 nm using a nanophotometer (Implen).

### 4.10 Quantification of expansion factor

To quantify the expansion factors (Figures 1A–C), the Nikon confocal AX setup was used. This modality allows the capture of images with a maximum field of view of 8,192 x 8,192 pixels to find corresponding regions before and after the expansion process. A Plan APO 60x/1.24 oil immersion objective was used to image the non-expanded sample and a Plan APO 60x/1.27 water immersion objective was used for the expanded samples. The microscope is equipped with an AX R Galvano scan head and a 2K resonant scanner. A DUX-VB detector unit with four GaAsP PMTs was used for confocal imaging. The AF488 fluorophore was excited using 12% of the 488 nm DPSS laser. The built-in dye preset of Nikon's NIS-Elements C software was used to select the appropriate excitation wavelength and filter settings for the specific dye.

### 4.11 General concept of cross-correlation analysis

The general concept of the algorithm is shown in Supplementary Figure 2. In the first step the region of interest (ROI) covering the synapse is detected. For this ROI a bounding box and an orientation of the major axis are computed. Subsequently, we compute an affine transform to align pre- and post-synapse. Here, we disable the scaling and apply a higher weighting to the translation parameters. To identify whether the data was sufficiently aligned we computed the Pearson correlation coefficient. A good correlation computed over the whole synaptic domain is indicative for the presence of nanocolumns (Figure 2D and Supplementary Figures 2–5). To check the corresponding correlation, we compute the dot product of the synapse orientation and shift vector. A result close to zero indicates a corresponding signal from the postsynaptic protein to its presynaptic counterpart.

### 4.12 Identifying pre- and postsynaptic structures

Images are processed with Otsu thresholding converting them into a binary format (Otsu, 1979). We further use sk-image (van der Walt et al., 2014) label to distinguish connected regions. These regions are sorted by size in descending order. We compute the region properties of the first element (the largest connected structure), i.e., orientation, major_axis, minor_axis, and bounding_box. Synapses are selected for analysis if the largest region is smaller than 300 pixel or has a major_axis/minor_axis < 2. For synapses meeting these criteria we try to connect the three largest signal clusters and test these properties again. If the region still fails, the synapse is discarded (Supplementary Figure 4). However, we saved a plot of the regions to verify the decision by hand. If the synapse is meeting the criteria mentioned above it is finally selected for analysis by the scientist performing the data evaluation. We save the evaluated properties.

Passing synapses are further processed with elastix. Here, the key component is the underlying parameter file.

### 4.13 Choice of parameter files

We used the “AffineDTITransform” for our parameter file due to its ability to give the degrees of freedom of the transform a weighting. To determine nanocolumns we expect the signal of the two channels to shift with a vector perpendicular to the major_axis of the pre- or postsynaptic structure. For example, the translation parameters therefore have the lowest parameter (Marstal et al., 2016), yielding the highest adjustability. The transform tries to transform in the following order: optimize translation -> rotation -> shear. The scaling is penalized by an order of magnitude that excludes it from the possible degrees of freedom.

It is also worth mentioning that the transform uses the geometrical image center as an initial point for the rotation. All other used parameters can be seen in the corresponding file in our repository (https://github.com/super-resolution/Sachs-et-al-2023-supplement) and are well-documented in the elastix manual.

We use the estimated transform to create a distortion map, showing the difference between non-aligned and aligned images with a distortion/vector map (Trinks et al., 2021). We save an overlay of the aligned images, the images before alignment together with the distortion map and data.txt, a file containing the file name, the Pearson correlation coefficient and the used image channels.

### 4.14 Colocalization analysis

For colocalization analysis of LGI1 with his synaptic partners, we focused on the LGI1 signal to maintain a stable orientation of the synapses. We analyzed the region of interest in Figure 4 with an automated Pearson correlation index and an image output of overlapping signals. All other used parameters can be seen in the corresponding file in our repository (https://github.com/super-resolution/Sachs-et-al-2023-supplement).

## Data availability statement

The original contributions presented in the study are included in the article/Supplementary material, further inquiries can be directed to the corresponding author.

## Ethics statement

The animal study was approved by Bavarian State authorities, Government of Unterfranken. The study was conducted in accordance with the local legislation and institutional requirements.

## Author contributions

SS: Conceptualization, Data curation, Validation, Visualization, Writing – original draft, Writing – review & editing, Formal analysis, Investigation, Methodology, Resources. SR: Data curation, Formal analysis, Investigation, Methodology, Validation, Visualization, Writing – original draft, Writing – review & editing, Software. JE: Investigation, Methodology, Validation, Visualization, Writing – review & editing. MS: Writing – review & editing, Conceptualization, Funding acquisition, Project administration, Supervision, Writing – original draft. CW: Conceptualization, Project administration, Supervision, Writing – original draft, Writing – review & editing, Data curation, Validation, Visualization.
