# Supplementary material for: Visualizing the trans-synaptic arrangement of synaptic proteins by expansion microscopy
Source: Front Cell Neurosci. 2024 Feb 29;18:1328726. doi: 10.3389/fncel.2024.1328726 (PMC10937466; doi:10.3389/fncel.2024.1328726)
Supplement: Supplementary file 1 [file Data_Sheet_1.DOCX]

Supplementary Material


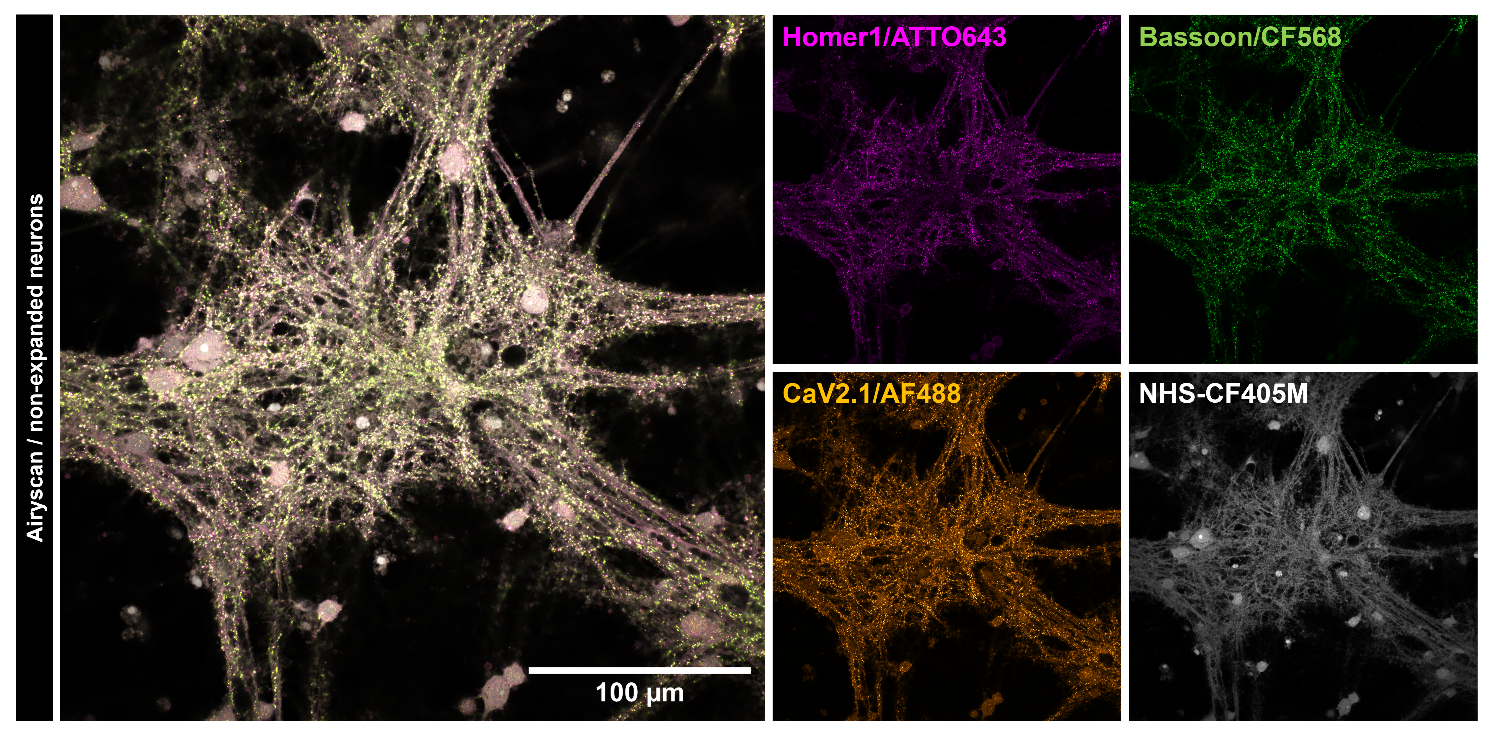


**Supplementary Figure 1.** **Synaptic protein organization visualized by 4-color Airyscan-imaging of non-expanded hippocampal mouse neurons.**

Immunostaining reveals synaptic markers in a neuronal context: Postsynaptic Homer1 (magenta), presynaptic BSN (green), and P/Q voltage-dependent calcium channels (CaV2.1, orange). Structural details are highlighted by NHS-ester staining (gray). Scale bar: 100 µm.


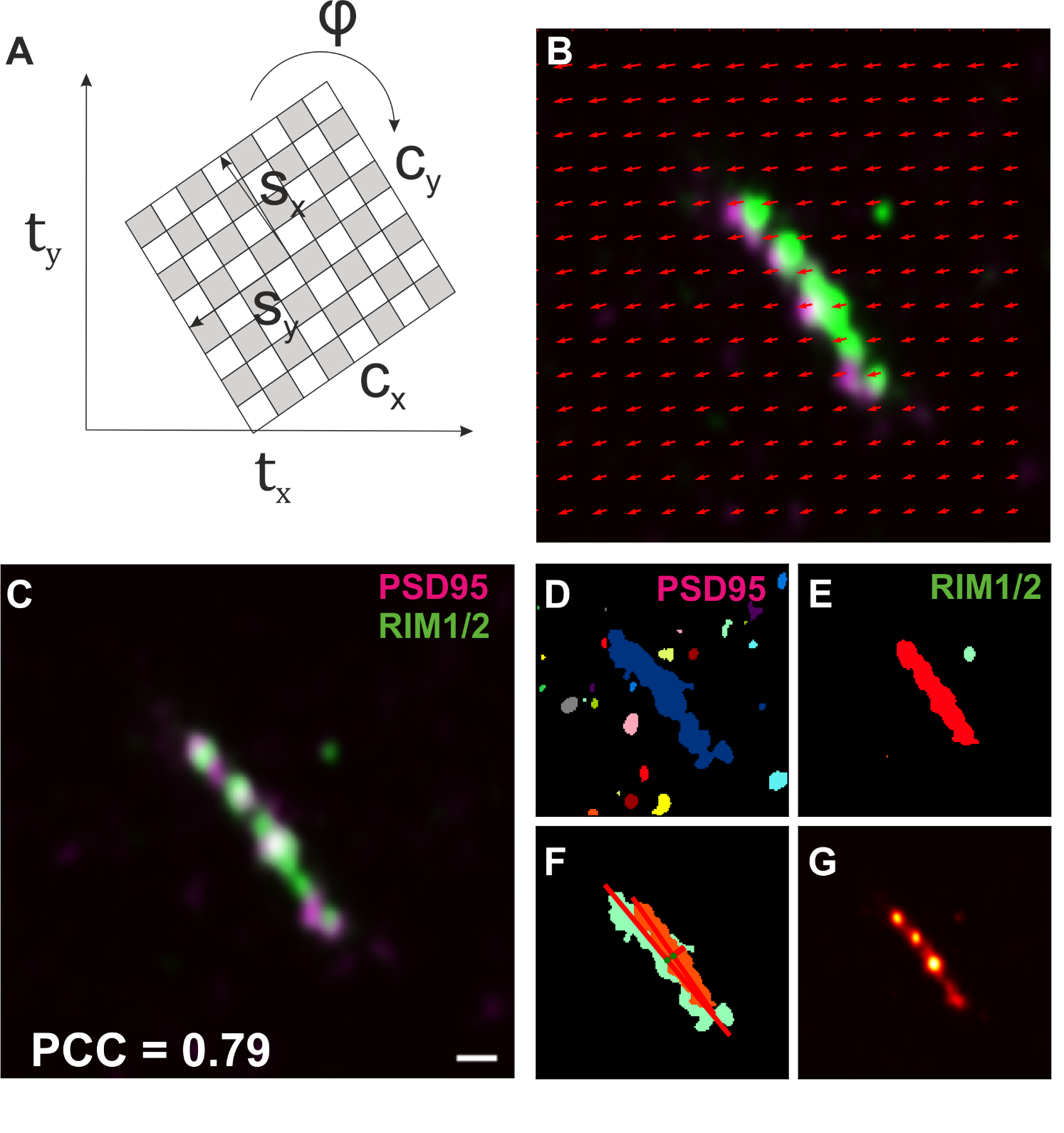


**Supplementary Figure 2. Computational evaluation of nanocolumns of PSD95 and RIM 1/2.**

**(A)** Affine transformation. **(B)** Distortion map indicating vectorial shifts for optimal alignment (red arrows). **(C)** Aligned image with high Pearson correlation value. **(D & E)** Image thresholding and labeling with a draggable threshold for user-defined region identification. **(F)** Representative regions of interest (ROI) are plotted with major axis and angle. **(G)** Multiplication of registered images enhances regions with correlated signals. Scale bar: 0.5 µm in ~8x expanded dimensions.


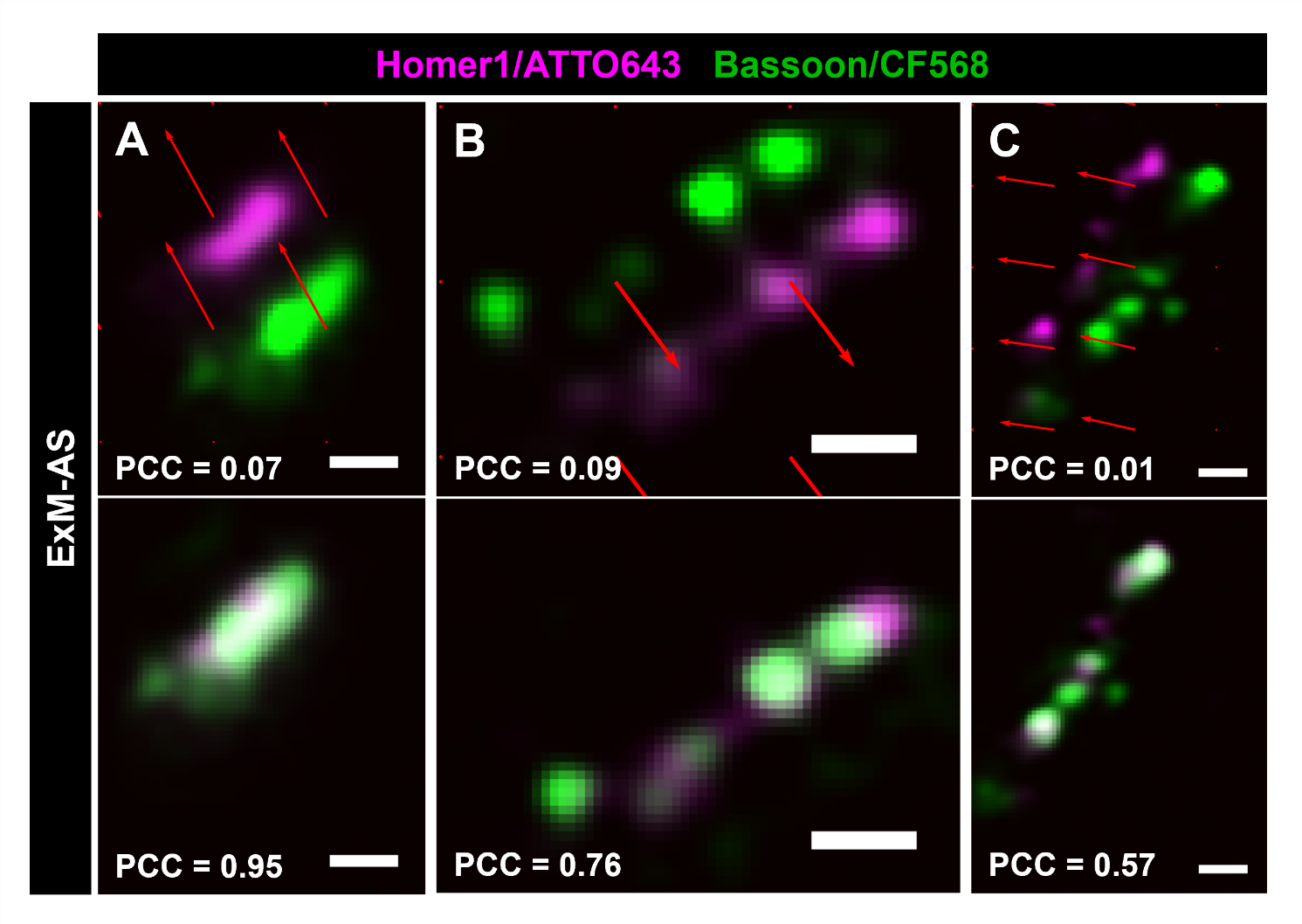


**Supplementary Figure 3. Representative examples of Bassoon and Homer1 nanocolumn alignment.**

Validation of Bassoon (green) and Homer1 (magenta) alignment is demonstrated in **(A-C)**. Red arrows in the upper part depict the vectorial shift of the distortion map. The lower part visualizes the overlap of aligned signals. Scale bars: 0.5 µm in ~8x expanded dimensions.


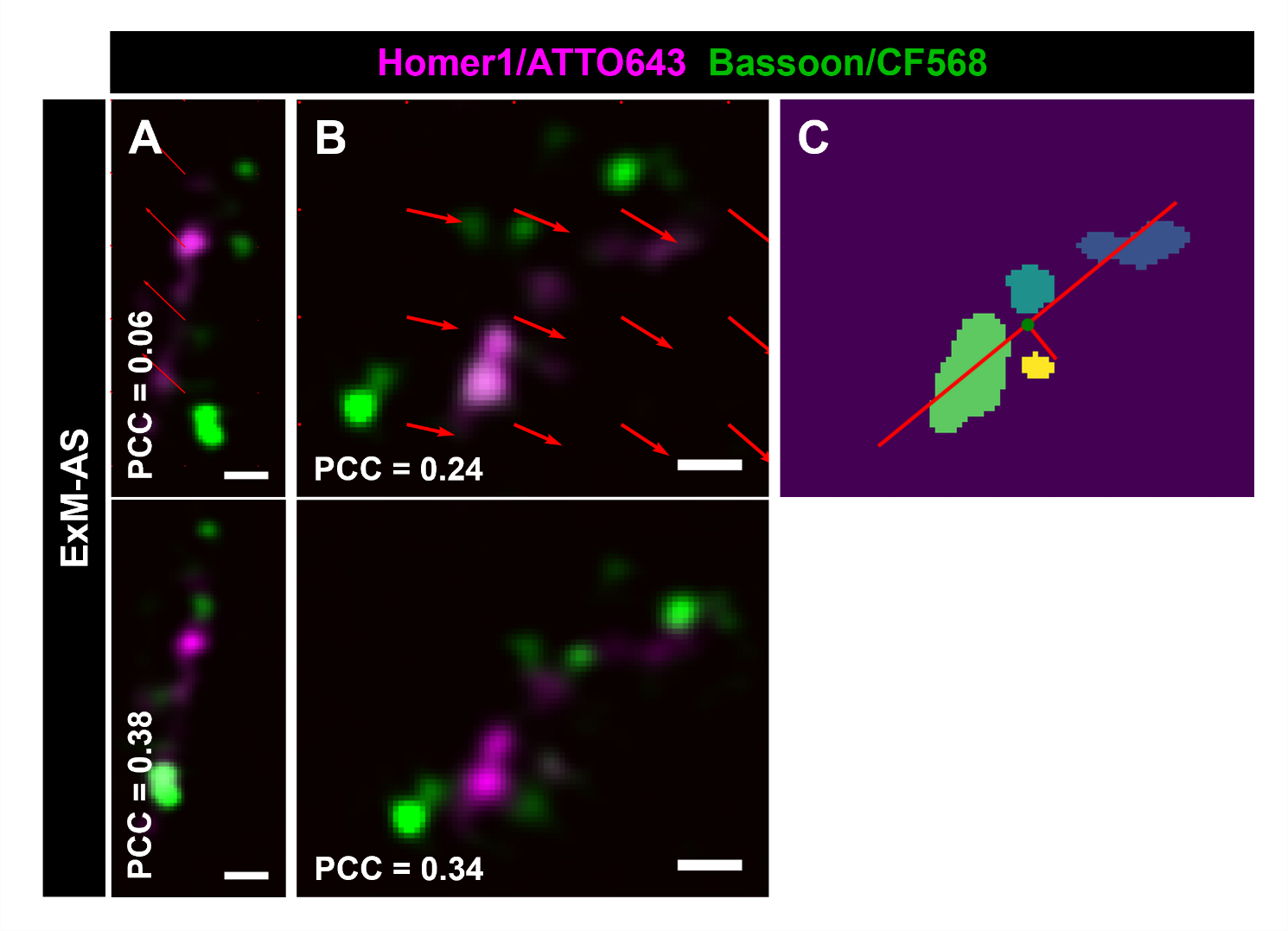


**Supplementary Figure 4**. **Excluded Homer1/Bassoon data examples.**

Presynaptic Bassoon (green) and postsynaptic Homer1 (magenta) alignment challenges, such as sparse signals, missing counterparts, and synapse bending. **(A)** Non-continuous Bassoon signal results in low PCC values. **(B)** Non-planar synapse orientation. (A-B) Red arrows indicating rotational correction (distortion map) **(C)** Homer1's geometrical center (green dot) and Bassoon's half-axis shift. These images were excluded from analysis (Figure 2E). Scale bars: 0.5 µm in ~8x expanded dimensions.


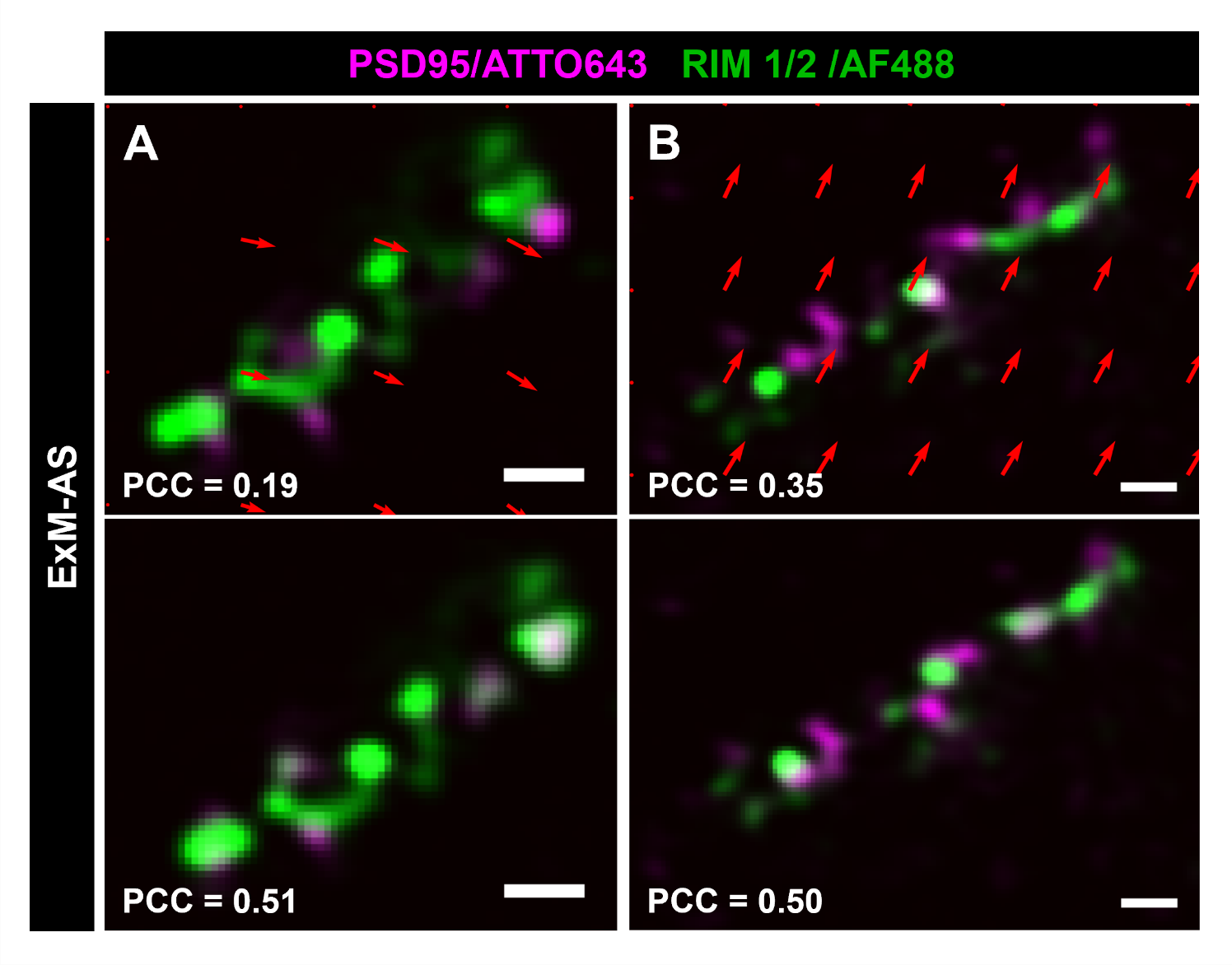


**Supplementary Figure 5**. **Illustration of RIM 1/2 and PSD95 nanocolumn alignment examples.** **(A & B)** Alignment of presynaptic RIM 1/2 (green) and postsynaptic PSD95 (magenta). The lower row displays the overlay of the aligned signals, and red arrows in the distortion map indicate the vectorial shift. Scale bars: 0.5 µm in ~8x expanded dimensions.


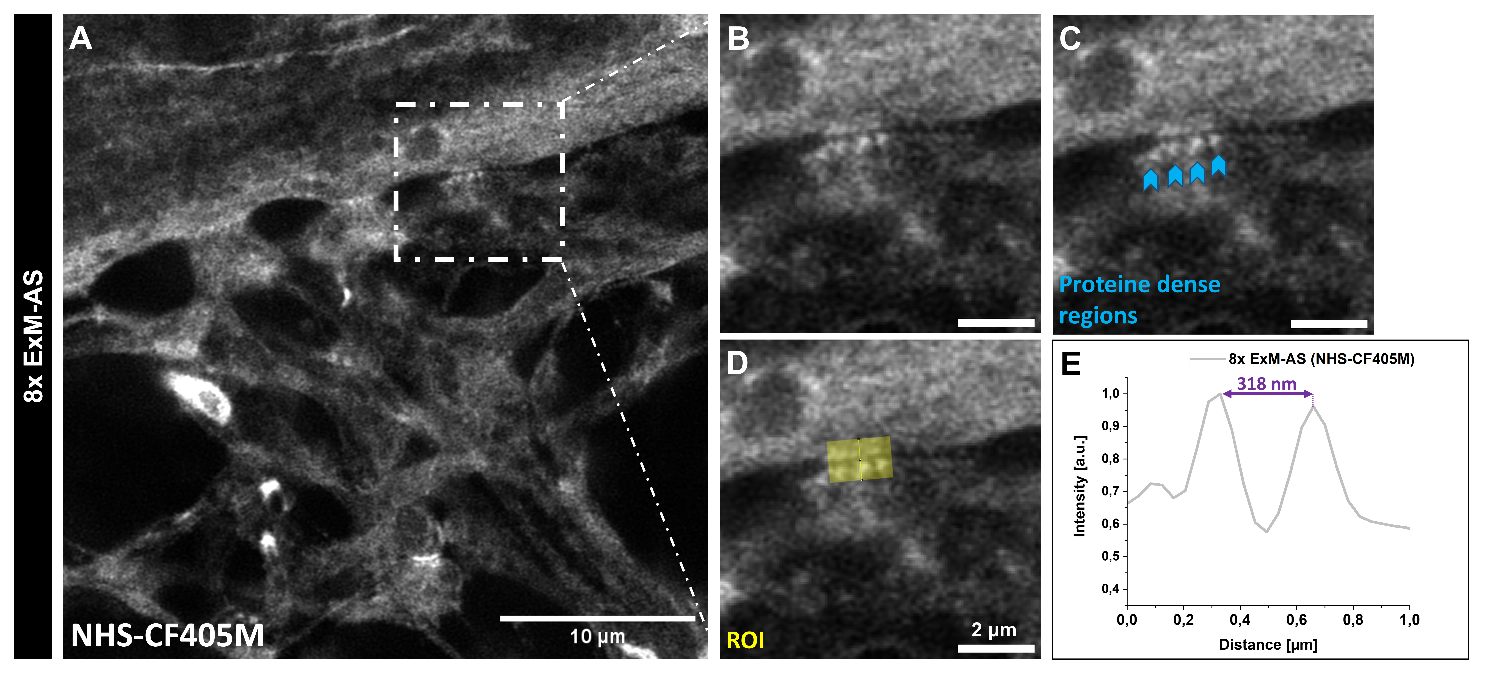


**Supplementary Figure 6.** **pan-ExM reveals the ultrastructural context of synapses.**

**(A)** Representative ExM-AS image (single slice of a 3D z stack) of ~8x expanded neuronal proteome. **(B)** Magnified view of the boxed region in (A). **(C)** NHS-staining visualizes dense protein networks marked by blue arrows. **(D)** ROI (yellow) selected for analysis of NHS-signal. **(E)** Representative plot showing normalized fluorescence intensity in a cross section of a synapse selected from (D, yellow). Peak to peak distance is 318 nm. (B-D) Scale bars: 2 µm in ~8x expanded dimensions.

**Software:**

DISCLAIMER

We provide the full evaluation software and processed data to guarantee full reproducibility of our results. We describe the software and the evaluation process in detail. Each evaluated output file can be viewed with the corresponding processing configurations.

We use SimpleElastix (Marstal et al., 2016) built from source. Since the repository is not maintained anymore, we provide the wheels for Python 3.6 and 3.10. If readers require wheels for another Python distribution, we encourage them to reach out to us, since the built process can be difficult and time-consuming due to outdated dependencies.

CONFIGURATION YAML FILES

The initially acquired images were distinguished into three categories:

**Best:** images with good continuous signals in both channels

**Stack:** images with synapses that extend over several z-slices. These are further processed under an average intensity projection.

**Worst:** images with non-continuous signal missing significant counterparts on one side of the synapse or including a lot of background, are therefore unsuitable for the following processing steps.

The processing pipeline is initialized with the processing tool hydra (Yadan, 2019). The corresponding configuration (.yaml) files contain the following parameters.

**Directory:**

**Condition:** Acquisition conditions of the file

**Culture:** Culture of the file

**Root_dir:** path to the root directory of the file

**Save name:** save name of the result file (defaults to filename + processing operation)

**Params:**

**Channels:** channels to compare

**Z_project:** indicates whether to perform an average intensity projection on the first dimension

**Transformation:**

**Type:** type of transformation that should be performed (can be affine, b-spline, similarity)

**Parameter_file:** path to the parameter file for the given transformation

**Type:** The type of transformation that should be applied. (We used a special affine transform)

**Parameter file:** Path to the parameter file for the elastix (Klein et al., 2010) alignment

COMPUTING STATISTICS

In our statistics, we iterate overall output folders and add all significant data as a column into a data frame. These can subsequently be plotted as a boxplot evaluating the condition of interest as well as being sorted by a column like the underlying culture. The data contains:

{Pearson, orientation, rotation, translation, culture, condition, category (Best, stack, worst)}
