# Supplementary material for: Visualizing the trans-synaptic arrangement of synaptic proteins by expansion microscopy
Source: Front Cell Neurosci. 2024 Feb 29;18:1328726. doi: 10.3389/fncel.2024.1328726 (PMC10937466; doi:10.3389/fncel.2024.1328726)
Supplement: Supplementary file 2 [file Table_1.DOCX]

**Table 1. Antibodies used for immunostaining**

Compilation of used antibodies for ExM-AS imaging.

| Antibody | Host | Working concentration | Product no. |
| --- | --- | --- | --- |
| *Primary antibodies* |  |  |  |
| PSD95 | mouse | 2 µg/ml | 124 011 Synaptic Systems |
| RIM 1/2 | guinea pig | 10 µg/ml | 140 205 Synaptic Systems |
| Homer1 | rabbit | 5 µg/ml | 160 003 Synaptic Systems |
| Bassoon | mouse | 5 µg/ml | 141 111 Synaptic Systems |
| CaV2.1 | guinea pig | 5 µg/ml | 257 185 1  Frontier Institute |
| Munc13-1 | guinea pig | 5 µg/ml | 126 115 Synaptic Systems |
| LGI1 | mouse | 10 µg/ml | MA5 27652 Invitrogen |
| GluA1 | rabbit | 5 µg/ml | ab86141 Abcam |
| GluA1 | rabbit | 7 µg/ml | ab31232 Abcam |
| *Secondary Antibodies* |  |  |  |
| guinea pig AF488 | goat | 10 µg/ml | 106 546 003 Jackson |
| mouse CF568 | goat | 10 µg/ml | SAB4600312 Sigma |
| rabbit ATTO643 | goat | 10 µg/ml | Self-labeled |

Compilation of used dyes for secondary antibody conjugation and pan-ExM.

| Antibody | Host | Working concentration | Product no. |
| --- | --- | --- | --- |
| *NHS-Dyes* |  |  |  |
| CF405M-NHS |  | 40 µg/ml | 92111 Biotium |
| ATTO643-NHS |  | 10 µg/ml | AD643 ATTO-Tec |

Compilation of used antibodies for *d*STORM imaging.

| Antibody | Host | Working concentration | Product no. |
| --- | --- | --- | --- |
| *Primary antibodies* |  |  |  |
| RIM 1/2 | guinea pig | 5 µg/ml | 140 205 Synaptic Systems |
| Homer1 | rabbit | 5 µg/ml | 160 003 Synaptic Systems |
| Bassoon | mouse | 5 µg/ml | 141 111 Synaptic Systems |
| PSD95 | mouse | 4 µg/ml | 124 011 Synaptic Systems |
| *Secondary Antibodies* |  |  |  |
| mouse CF568 | goat | 5 µg/ml | SAB4600312 Sigma |
| guinea pig CF568 | donkey | 5 µg/ml | SAB4600469 Sigma |
| mouse AF647 | goat | 5 µg/ml | A21237 Invitrogen |
| rabbit AF647 | goat | 5 µg/ml | A21246 Invitrogen |

Compilation of used antibodies for expansion factor quantification

| Antibody | Host | Working concentration | Product no. |
| --- | --- | --- | --- |
| *Primary antibodies* |  |  |  |
| Neurofilament L | mouse | 10 µg/ml | 171 011 Synaptic Systems |
| *Secondary Antibodies* |  |  |  |
| mouse AF488 | mouse | 10 µg/ml | A11017 Invitrogen |
